# Overexpression of the Mitochondrial T3 Receptor Induces Skeletal Muscle Atrophy during Aging

**DOI:** 10.1371/journal.pone.0005631

**Published:** 2009-05-20

**Authors:** François Casas, Laurence Pessemesse, Stéphanie Grandemange, Pascal Seyer, Olivier Baris, Naïg Gueguen, Christelle Ramonatxo, Florence Perrin, Gilles Fouret, Laurence Lepourry, Gérard Cabello, Chantal Wrutniak-Cabello

**Affiliations:** 1 INRA, UMR866 Différenciation cellulaire et croissance, Montpellier, France; 2 Université Montpellier I et II, Montpellier, France; 3 INSERM, U583 Institut des Neurosciences de Montpellier, Université Montpellier I et II, Montpellier, France; University of Las Palmas de Gran Canaria, Spain

## Abstract

In previous studies, we characterized a new hormonal pathway involving a mitochondrial T3 receptor (p43) acting as a mitochondrial transcription factor. In *in vitro* and *in vivo* studies, we have shown that p43 increases mitochondrial transcription and mitochondrial biogenesis. In addition, p43 overexpression in skeletal muscle stimulates mitochondrial respiration and induces a shift in metabolic and contractile features of muscle fibers which became more oxidative.

Here we have studied the influence of p43 overexpression in skeletal muscle of mice during aging. We report that p43 overexpression initially increased mitochondrial mass. However, after the early rise in mitochondrial DNA occurring at 2 months of age in transgenic mice, we observed a progressive decrease of mitochondrial DNA content which became 2-fold lower at 23 months of age relatively to control animals. Moreover, p43 overexpression induced an oxidative stress characterized by a strong increase of lipid peroxidation and protein oxidation in quadriceps muscle, although antioxidant enzyme activities (catalase and superoxide dismutase) were stimulated. In addition, muscle atrophy became detectable at 6 months of age, probably through a stimulation of the ubiquitin proteasome pathway via two muscle-specific ubiquitin ligases E3, Atrogin-1/MAFbx and MuRF1.

Taken together, these results demonstrate that a prolonged stimulation of mitochondrial activity induces muscle atrophy. In addition, these data underline the importance of a tight control of p43 expression and suggest that a deregulation of the direct T3 mitochondrial pathway could be one of the parameters involved in the occurrence of sarcopenia.

## Introduction

Triiodothyronine (T3) exerts a pleiotropic influence on development and homeostasis of the adult. In particular, this hormone is considered as an important regulator of muscle development. Not only does it stimulate growth by increasing the number and diameter of muscle fibers [1], [2], but it also regulates the transition between neonatal and adult myosin isoforms [3] and the contractile features of adult muscle fibers [4]. Thyroid hormone acts through nuclear receptors (T3Rs) encoded by TRα and TRβ genes (NR1A1 and NR1A2 according to nuclear hormone receptor nomenclature) [5], [6]. These receptors are ligand-dependent transcription factors that constitutively bind to specific sequences called thyroid hormone response elements (T3RE) located in the promoter of T3 target genes. We have previously identified in mitochondria truncated forms of the nuclear receptor TRα1, with molecular weights of 43 kDa (p43) and 28 Kda [7], [8]. P43 is a mitochondrial T3 receptor which stimulates mitochondrial transcription and protein synthesis in the presence of T3 [9]. In C2C12 cells, its overexpression stimulates mitochondrial activity and potentiates terminal differentiation [10], [11]. More recently, in *in vivo* studies, we have shown that p43 overexpression in skeletal muscle increases mitochondrial transcription and mitochondrial biogenesis inducing a stimulation of mitochondrial respiration and changes in metabolic and contractile features of muscle fibers which became more oxidative [12]. In the oxidative muscle soleus, MyHC IIa fibers were partly replaced by type I fibers; in the oxido-glycolytic muscle quadriceps, the frequency of MyHC IIa and IIx fibers was increased in association with a reduction in the number of glycolytic IIb fibers. In parallel in *in vitro* studies, we reported that overexpression of p43 in human dermal fibroblasts stimulates ROS production [13]. These data suggest that p43 overexpression in skeletal muscle could also induce a similar event, hence inducing an oxidative stress which might contribute to muscle wasting via proteolysis activation.

Here we report that p43 overexpression in skeletal muscle induces an oxidative stress despite stimulation of antioxidant enzyme activities. In addition, this oxidative stress induces skeletal muscle atrophy detectable at 6 months of age, concomitant to a stimulation of the ubiquitin proteasome pathway involving two muscle-specific ubiquitin ligases E3, Atrogin-1/MAFbx [14] and MuRF1 [15] whose expression is strongly increased.

## Results

### Influence of p43 overexpression on mitochondrial function during aging

**Figure 1:**
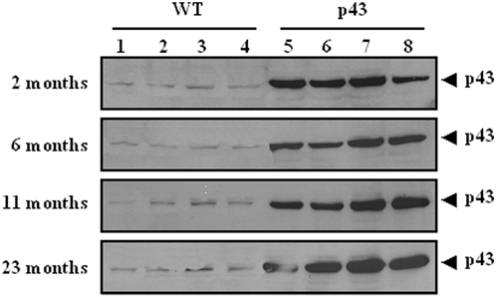
p43 overexpression during aging. P43 protein levels in quadriceps muscle extracts from transgenic mice versus wild-type animals, assessed by western-blot using an antibody raised against TRα. 50 µg of total protein extracts were analyzed.

We have first verified that p43 was actually overexpressed throughout the life of transgenic animals in western-blot experiments using quadriceps muscle extracts. We confirmed that p43 overexpression was easily detected in muscle extracts from mouse at 2, 6, 11 and 23 months of age (Figure 1).

**Figure 2:**
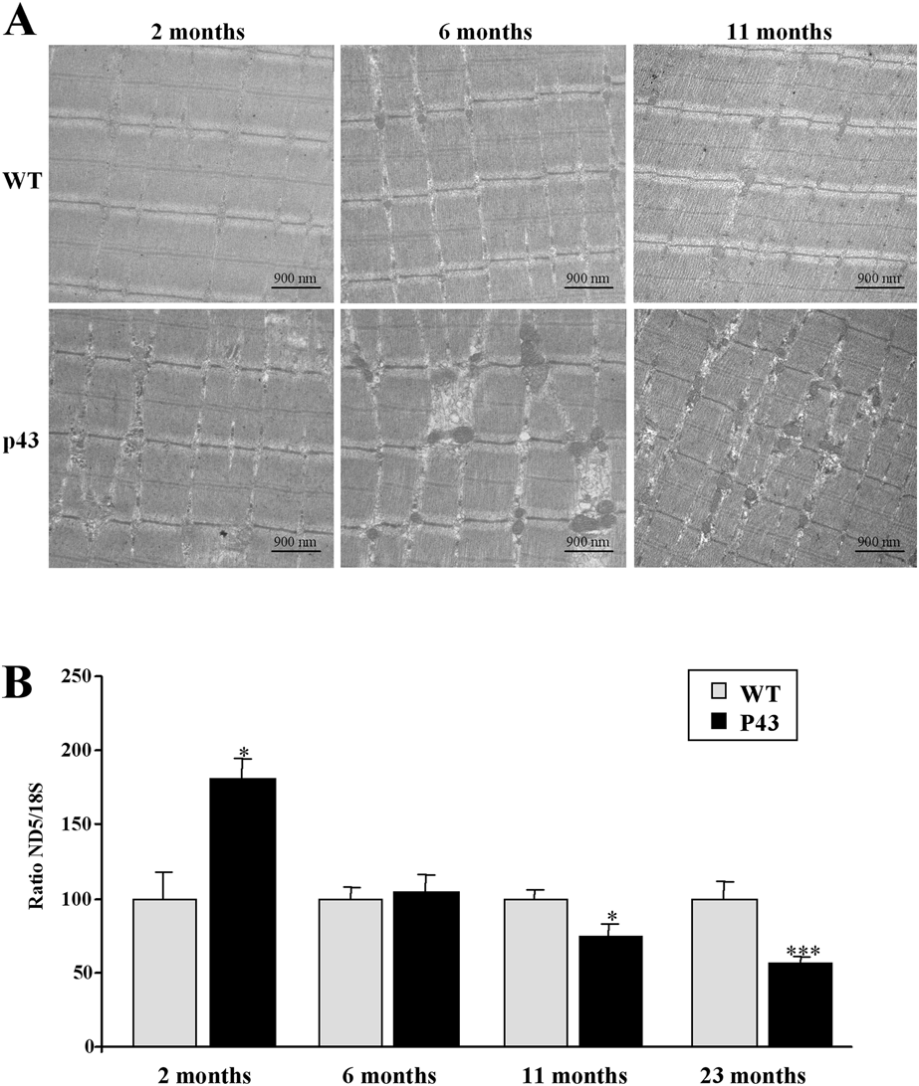
Influence of p43 overexpression during aging on mitochondrial biogenesis. (A) electron microscopy experiments from longitudinal sections taken from quadriceps from transgenic mice (p43) and wild-type animals (WT) at 2, 6 and 11 months of age (magnification ×10.000). (B) Relative mtDNA content in quadriceps muscle from transgenic mice (p43) compared to wild-type animals (WT) at 2, 6, 11 and 23 months of age (n = 8 for each group with the exception of 23 month old-group where n = 6). After extraction of muscle DNA, quantitative PCR reactions were performed using ND5 for mtDNA copy estimation, and 18S for the nuclear genome. *p<0.05 and ***p<0.001. Control values at each stage are considered as 100%, and p43 value are expressed in percent of the corresponding control value.

We then studied changes in mitochondrial activity and mitochondrial biogenesis in skeletal muscle of 2, 6, 11 and 23 months old mice. In electron microscopy studies performed on quadriceps muscle from 2, 6 and 11 months old mice, p43 overexpression increased mitochondrial mass and organelle size whatever the stage (Figure 2A). In addition, we have assessed the amount of mitochondrial DNA (mt-DNA) in this muscle, assessed by the value of the ratio of mitochondrial ND5 gene level relatively to nuclear 18S gene level. The ratio ND5/18S was significantly higher in transgenic mice than in wild type mice at 2 months of age (+81%, p<0.05), but progressively decreased during aging to be 2-fold lower at 23 months of age (−44%, p<0.001) (Figure 2B).

**Figure 3:**
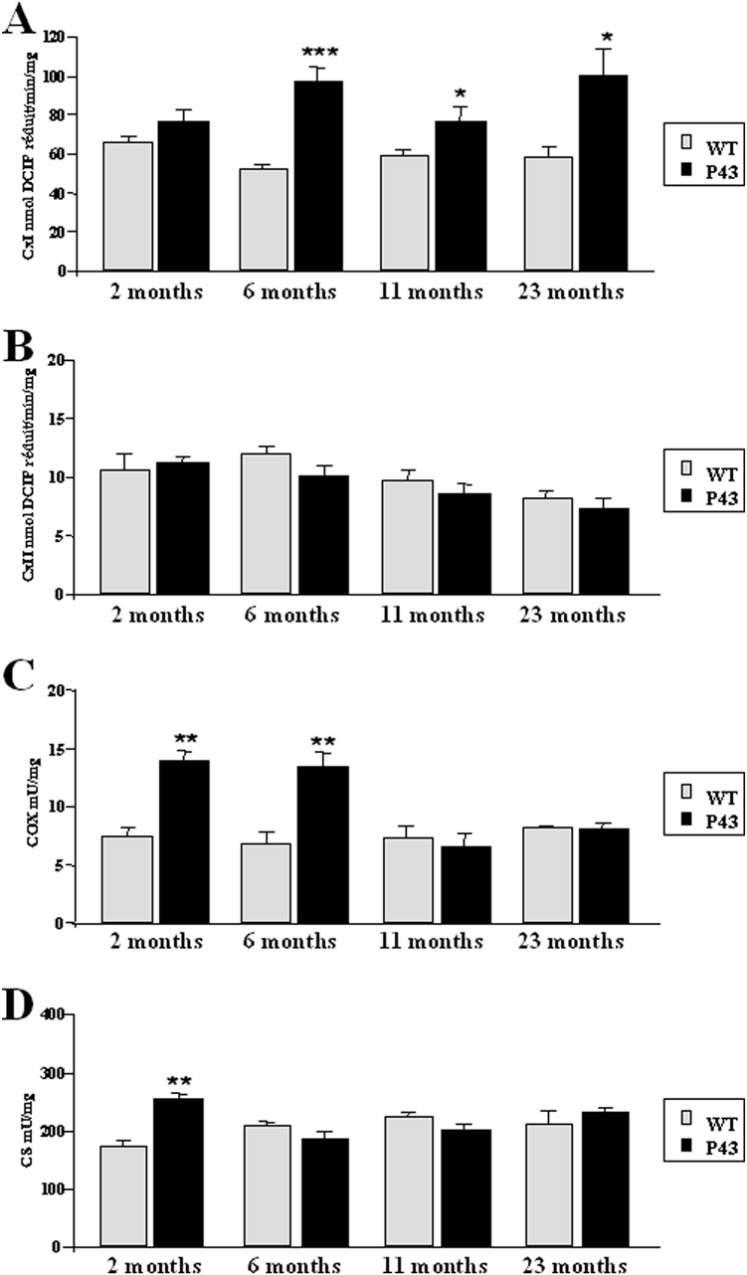
Influence of p43 overexpression during aging on mitochondrial activity. (A–D) Complex I (CXI), Complex II (CXII), Cytochrome oxidase (COX), and Citrate Synthase (CS) activities in gastrocnemius muscle extracts from transgenic mice (p43) and wild-type animals (WT) at 2, 6, 11 and 23 months of age (n = 8 for each group with the exception of 23 month old-group where n = 6). *p<0.05, **p<0.01 and ***p<0.001.

In gastrocnemius of control mice, we did not record any age-related changes in complexes I and II, cytochrome oxidase (COX) and citrate synthase (CS) maximal activity (Figure 3). P43 overexpression strongly stimulated COX activity at 2 and 6 months (p<0.01 at both stages), but this stimulation was no longer detected in 11 and 23 months old mice (Figure 3C). Citrate synthase activity was only increased in 2 months old transgenic mice (+46%; p<0.01), and remained similar to that recorded in control animals thereafter (Figure 3D). In p43 overexpressing mice, complex I activity was higher at 6, 11 and 23 months than in control ones (respectively p<0.001, p<0.05 and p<0.05). However, this stimulation was not detected at 2 months (Figure 3A). Activity of complex II, a complex including only nuclear encoded subunits, was not modified whatever the age of mice (Figure 3B).

**Figure 4:**
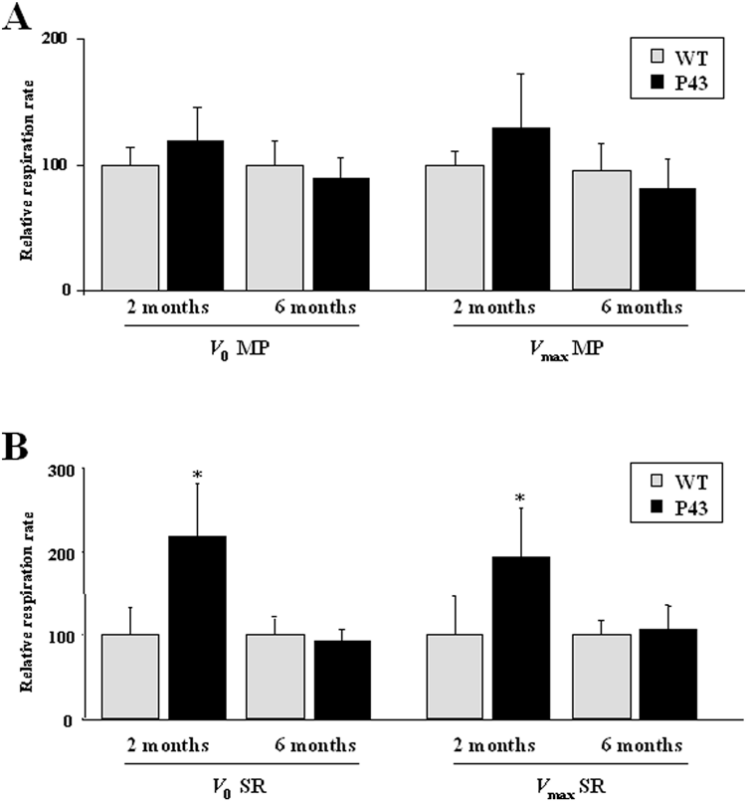
Influence of p43 overexpression on mitochondrial respiration at 2 and 6 months of age. (A–B) Relative respiration rate in permeabilized gastrocnemius muscle fibers from transgenic mice (p43) and wild-type animals (WT) at 2 and 6 months of age (n = 5 in each group). Respiration parameters were recorded at 25°C using an Oroboros oxygraph. Resting respiration (*V*_0_) was initiated in the presence of complex I (5 mM malate and 5 mM pyruvate) or complex II substrates (10 mM succinate and 2.5 µM rotenone), and maximal ADP-stimulated respiration was measured with one addition of saturating ADP concentration (1 mM)(*V*_max_). MP: malate and pyruvate; SR: succinate and rotenone. *p<0.05. Control values at each stage are considered as 100%, and p43 value are expressed in percent of the corresponding control value.

Oxygen consumption was measured on isolated permeabilized fibers from gastrocnemius muscle in 2 and 6 months old animals. Resting respiration (*V*_0_) was assessed in the presence of complex I (malate/pyruvate) or complex II substrates (succinate/rotenone), and maximal ADP-stimulated respiration (*V*_max_) was measured with addition of saturating ADP concentration. In the presence of complex I substrates no significant differences were recorded (Figure 4A) between transgenic and control mice. In the presence of complex II substrates, an up to two-fold increase of *V*_0_ and *V*_max_ (+92%) respiration rate was recorded in 2-months old transgenic mice whereas no difference remained at 6 months of age (Figure 4B).

According to this set of data, p43 overexpression induced a transient rise in mitochondrial biogenesis and activity detected in 2 and/or 6 months old animals, according to the parameter measured, followed by a progressive decrease leading to values similar (respiration, COX, CS) or even lower that recorded in control animals (mt-DNA).

### Influence of p43 overexpression on muscle oxidative stress

To test the possibility that the p43-induced stimulation of mitochondrial activity could lead to an oxidative stress as previously shown *in vitro* [13], we assessed lipid peroxidation, protein oxidation and antioxidant enzyme activities (superoxide dismutase and catalase) or other markers affected by oxidative stress (uncoupling proteins, UCP2, UCP3 mRNA) in quadriceps muscle extracts.

**Figure 5:**
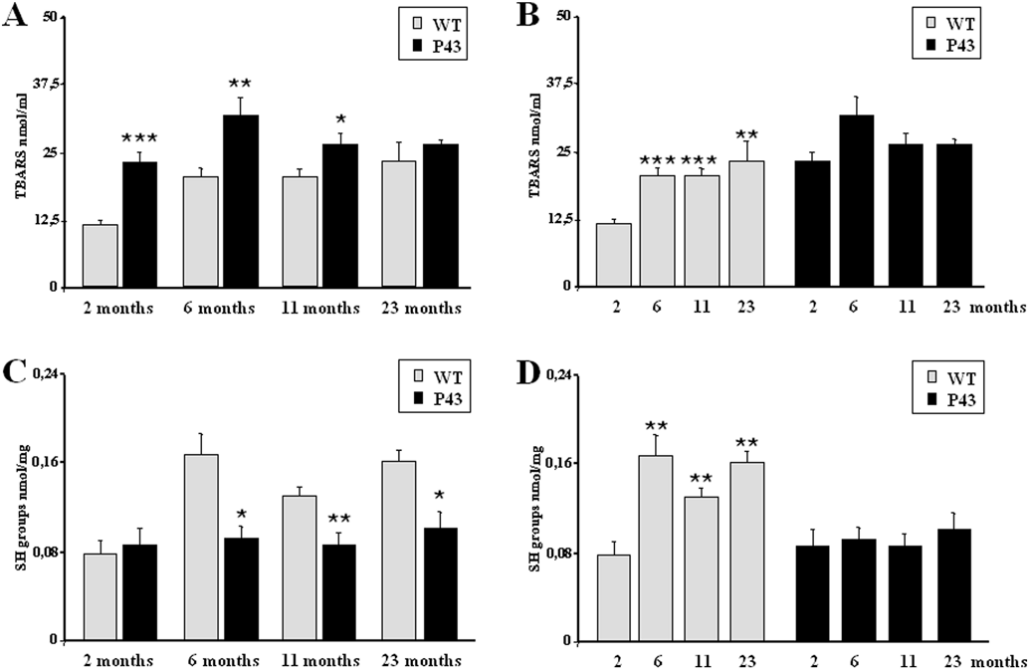
p43 overexpression induces a potent oxidative stress. (A–B) Concentration of the thiobarbituric acid-reactive substances (TBARS) in quadriceps muscle from transgenic mice (p43) and wild-type animals (WT) at 2, 6, 11 and 23 months of age (n = 8 for each group with the exception of 23 month old-group where n = 6). *p<0.05, **p<0.01 and ***p<0.001. (C–D) Concentration of thiol (SH-groups) in quadriceps from transgenic mice (p43) and wild-type animals (WT) at 2, 6, 11 and 23 months of age (n = 8 for each group with the exception of 23 month old-group where n = 6). *p<0.05 and **p<0.01.

Lipid peroxidation, assessed by measurement of thiobarbituric acid-reactive substances (TBARs), increased throughout life in control animals (Figure 5B). However, TBARs levels did not change from 2 to 23 months of life in transgenic mice (Figure 5B) and remained significantly higher than in control ones all over the first 11 months of life (respectively +102%, p<0.001; +55%, p<0.01; +29%, p<0.05) (Figure 5A). Oxidative damage to proteins is noticable by a decrease in protein thiols (SH groups) levels. SH groups increased from 2 to 6 months in control animals, thus suggesting reduced amounts of oxidized proteins in older animals (Figure 5D). P43 overexpression abrogated this rise (Figure 5D). As a consequence, the amounts of SH groups were lower in transgenic mice than in control ones from 6 to 23 months of life (respectively −46%, p<0.05; −35%, p<0.01; −40%, p<0.05) (Figure 5C).

**Figure 6:**
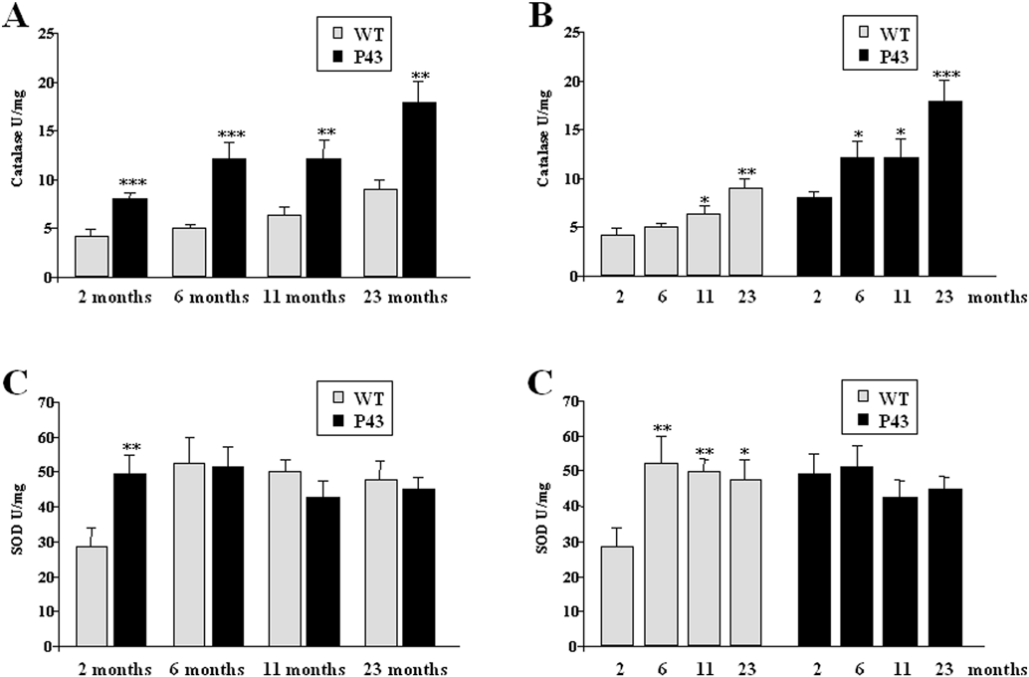
p43 overexpression increases antioxidants activities. (A–B) Catalase activity in quadriceps muscle from transgenic mice (p43) compared to wild-type animals (WT) at 2, 6, 11 and 23 months of age (n = 8 for each group with the exception of 23 month old-group where n = 6). (C–D) Superoxide dismutase (SOD) activity in quadriceps muscle from transgenic mice (p43) compared to wild-type animals (WT) at 2, 6, 11 and 23 months of age (n = 8 for each group with the exception of 23 month old-group where n = 6). **p<0.01 and ***p<0.001.

**Figure 7:**
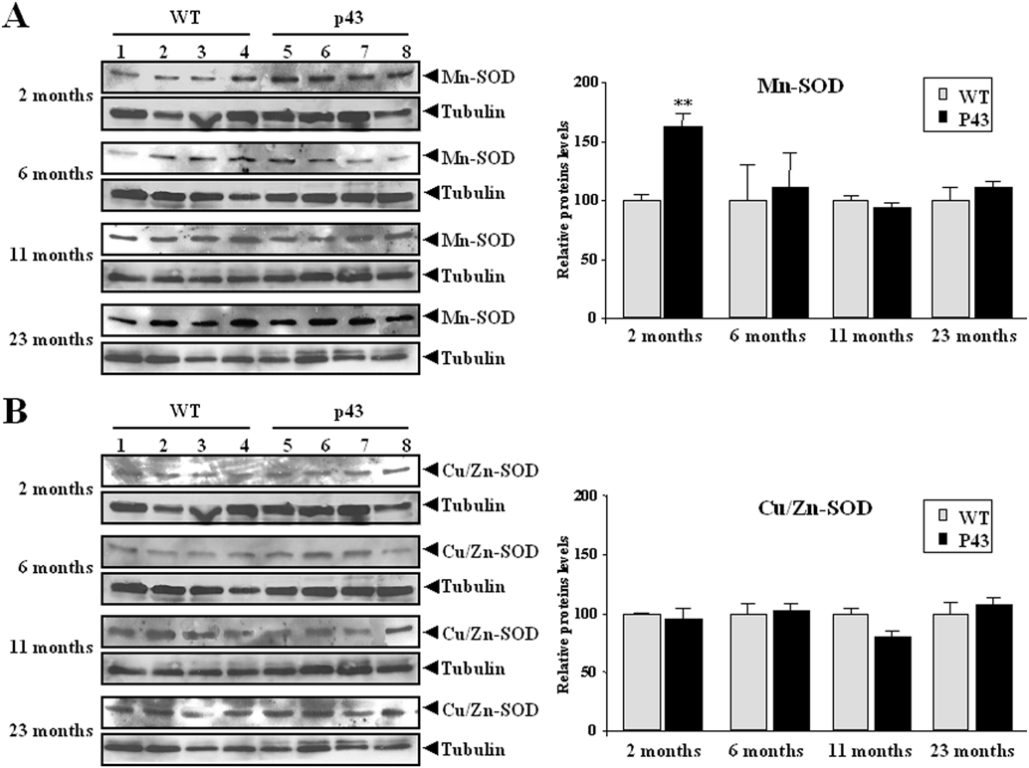
p43 overexpression increases MnSOD activity in 2 month-old transgenic mice. (A) Cu/ZN-SOD protein levels in quadriceps muscle extracts from transgenic mice (p43) and wild-type animals (WT), assessed by western-blot using an antibody raised against Cu/ZN-SOD. (B) Mn-SOD protein levels in quadriceps muscle extracts from transgenic mice (p43) and wild-type animals (WT), assessed by western-blot using an antibody raised against Mn-SOD. 50 µg of total protein extracts were analyzed. Relative protein levels were indicated. **p<0.01. Control values at each stage are considered as 100%, and p43 value are expressed in percent of the corresponding control value.

Antioxidant enzymes such as catalase and superoxide dismutase (SOD) are recruited to counteract free radical damage. In control animals their activity increased after 6 (SOD) or 11 months of life (catalase) (Figure 6B and D). A similar rise was recorded for catalase activity in p43 overexpressing mice, but this activity remained higher than in control animals throughout life (2 months: +92%, p<0.001; 6 months: +143%, p<0.001; 11 months: +89%, p<0.01; 23 months: +99%, p<0.01) (Figure 6A). In contrast, in the former, no age-related changes in SOD activity could be detected, and was only higher than in control animals 2 months after birth (+73%, p<0.01) (Figure 6C). Measurements of Mn-SOD and Cu-Zn/SOD expression by western-blot clearly suggested that this higher value at 2 months of age was essentially explained by a rise in Mn-SOD expression in transgenic mice (+65%, p<0.01) (Figure 7B).

**Figure 8:**
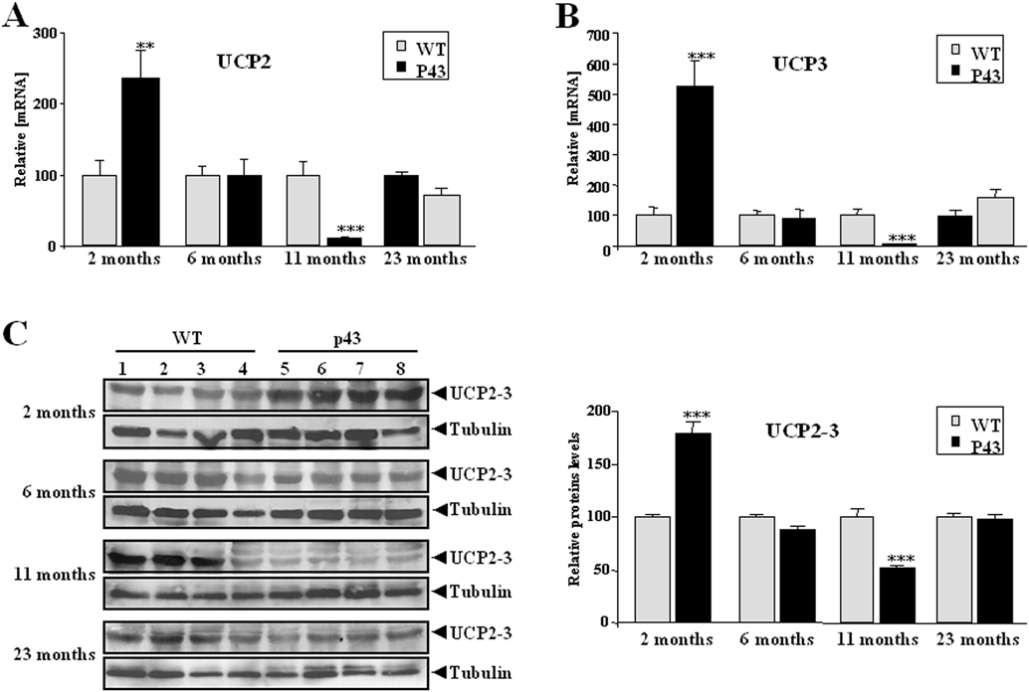
Influence of p43 overexpression during aging on UCP2 and UCP3 expression. (A–B) Relative mRNA expression levels of UCP2 and UCP3 in quadriceps muscle from transgenic mice (p43) and wild-type animals (WT) at 2, 6, 11 and 23 months of age (n = 8 for each group with the exception of 23 month old-group where n = 6). **p<0.01 and ***p<0.001. (C) UCP2 and UCP3 protein levels in quadriceps muscle extracts from transgenic mice (p43) and wild-type animals (WT), assessed by western-blot using an antibody raised against UCP2 and UCP3. 50 µg of total protein extracts were analyzed. Relative protein levels were indicated. **p<0.01 and ***p<0.001. Control values at each stage are considered as 100%, and p43 value are expressed in percent of the corresponding control value.

One of the main mitochondrial adaptations to oxidative stress is a mild uncoupling of oxidative phosphorylation that reduces the mitochondrial production of ROS by lowering mitochondrial membrane potential [16], [17]. We found that UCP2 and UCP3 mRNAs measured by quantitative PCR were strongly induced in quadriceps muscle extracts from 2-months old transgenic mice relatively to control ones (respectively +137%, p<0.01; +425%, p<0.001) (Figure 8A–B). In line with mRNA data, we showed that UCP2-3 proteins were strongly increased in quadriceps muscle extracts from 2-months old transgenic mice relative to controls (respectively +176%, p<0.001) (Figure 8C). Interestingly no difference was recorded in 6 and 23 month old animals, and a strong reduction in UCP2 and 3 mRNA expression in p43 overexpressing mice was even recorded at 11 months of age (respectively −90%, p<0.001 and −92%, p<0.001) (Figure 8A–B). These decrease at 11 months of age was confirmed at the protein level (−48%, p<0.00) (Figure 8C).

All these data suggest that the transient initial rise in mitochondrial activity induced by p43 overexpression led to a long term oxidative stress despite an increase in antioxidant enzyme activity.

### P43 influence on muscle development

**Figure 9:**
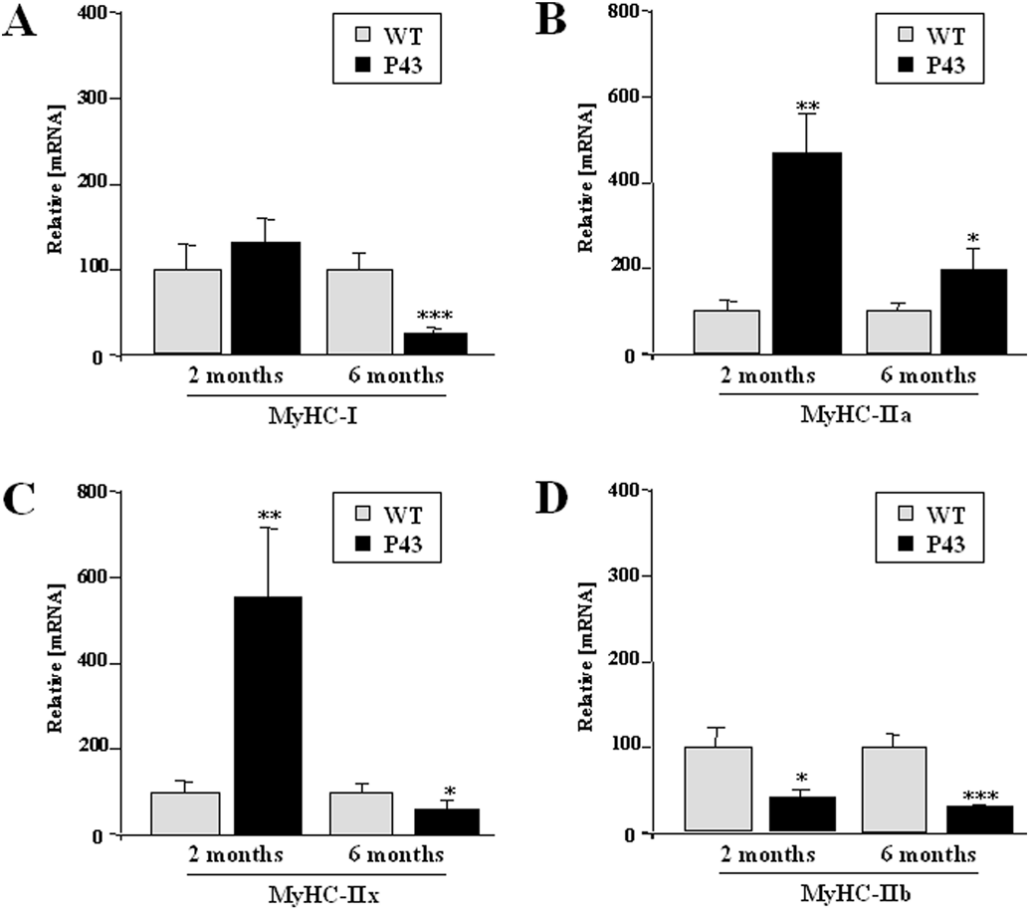
Influence of p43 overexpression on MyHC isoforms expression at 2 and 6 months of age. (A–D) Relative mRNA expression levels of the four adult MyHC isoforms in quadriceps muscle from transgenic mice (p43) and wild-type animals (WT) at 2 and 6 months of age (n = 8 each group). *p<0.05; **p<0.01, ***p<0.001. Control values at each stage are considered as 100%, and p43 value are expressed in percent of the corresponding control value.

To assess changes in contractile features of muscle fibres occurring during aging in control or p43 overexpressing mice, we studied the expression of the four adult MHCs transcripts in quadriceps (oxido-glycolytic muscle) collected from 2 and 6 months old mice. As previously shown [12], a shift toward a slower muscle phenotype was recorded in 2 months old transgenic mice, characterized by a rise in MyHCIIa and IIx expression and a simultaneous decrease in MyHCIIb expression (Figure 9). However, at 6 months of age, expression of MyHC-I and IIx decreased in transgenic mice (respectively −75%, p<0.001, −40%, p<0.05) (Figure 9A and 9C). In addition, though MyHCIIa transcript levels were significantly higher in p43 overexpressing mice at 6 months of age, we observed a decrease in this transcript level during aging (+467% at 2 months vs +96% at 6 months) (Figure 9B). Lastly, MyHCIIb transcript levels were lower in transgenic mice than in control mice at 2 and 6 months of age (respectively −58%; p<0.05 and −69%; p<0.001) (Figure 9D).

**Figure 10:**
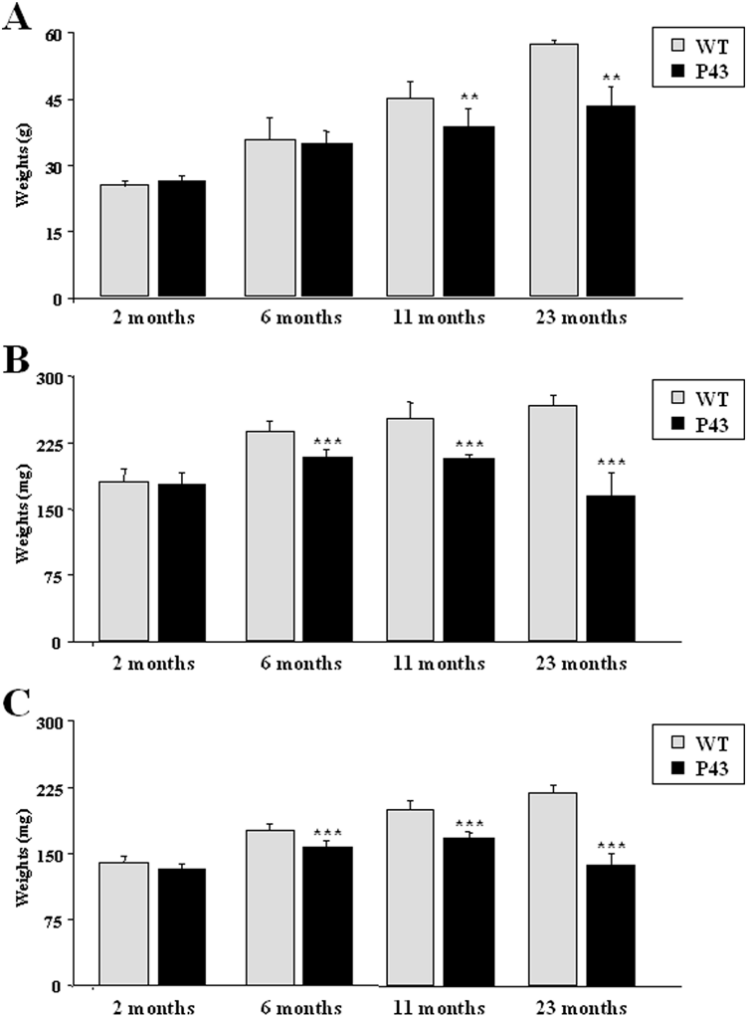
Body and muscle weights of wild-type and p43 transgenic male mice at 2, 6, 11 and 23 months of age. (A) Body weights recorded in transgenic mice (p43) and wild-type animals (WT) at 2, 6, 11 and 23 months of age (n = 8 for each group with the exception of 23 month old-group where n = 6). (B) Quadriceps muscle weights recorded in transgenic mice (p43) and wild-type animals (WT) at 2, 6, 11 and 23 months of age (n = 8 for each group with the exception of 23 month old-group where n = 6). (C) Gastrocnemius muscle weights recorded in transgenic mice (p43) and wild-type animals (WT) at 2, 6, 11 and 23 months of age (n = 8 for each group with the exception of 23 month old-group where n = 6). **p<0.01 and *** p<0.001.

In control mice, muscle weight increased progressively throughout life. This tissular growth was markedly reduced in transgenic animals, and muscle weight was significantly reduced in comparison to that recorded in control animals since 6 months of life (Figure 10). More striking was the observation that p43 overexpression induced an important loss of muscle mass in older animals (Figure 10). In addition, in 2, 6 and 11 months old transgenic mice, we observed structural abnormalities with an increased amount of interstitial tissue and glycogen accumulation; we also recorded a variability in muscle fiber size and an alteration of muscle structure (Figure 2A).

**Figure 11:**
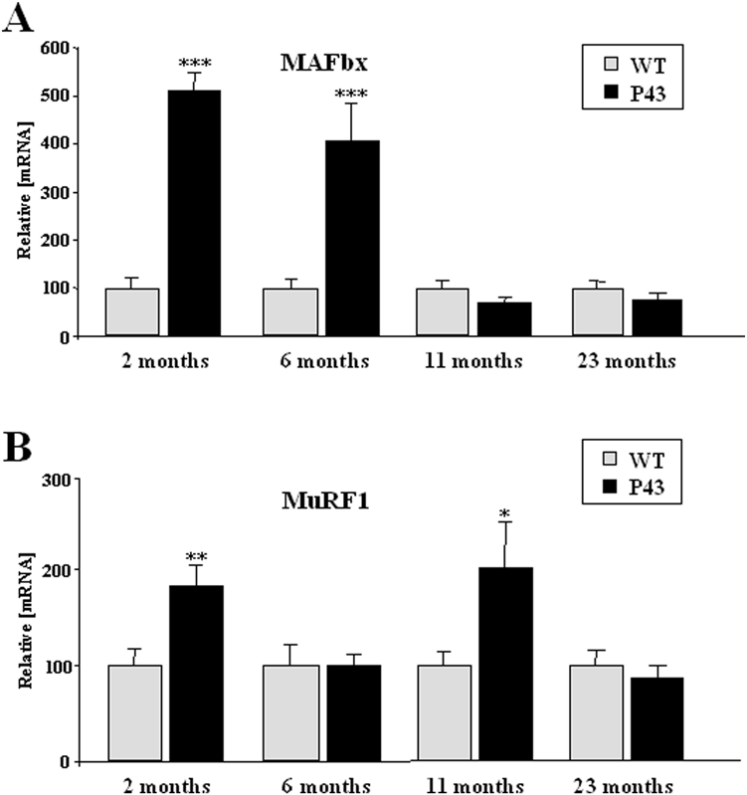
p43 overexpression increases MAFbx and MuRF1 expression. (A–B) Relative mRNA expression levels of MAFbx and MuRF1 in quadriceps muscle from transgenic mice (p43) and wild-type animals (WT) at 2, 6, 11 and 23 months of (n = 8 for each group with the exception of 23 month old-group where n = 6). *p<0.05, **p<0.01 and ***p<0.001. Control values at each stage are considered as 100%, and p43 value are expressed in percent of the corresponding control value.

Muscle proteolysis is known to be mediated by the ubiquitin proteasome pathway through two muscle-specific ubiquitin ligases E3: MAFbx (muscle atrophy Fbox or atrogin-1) [14] and MuRF1, (muscle ring finger) [15]. MAFbx and MuRF1 are critical regulators of the enhanced proteolysis leading to muscle atrophy in various diseases [18]. In agreement with the muscle mass loss observed in transgenic animals, MAFbx (2 and 6 months) and MuRF1 (2 and 11 months) expressions were significantly enhanced relatively to control mice (Figure 11). However, no differences remained between the two groups at a stage (23 months) where the more severe degree of sarcopenia occurred.

**Figure 12:**
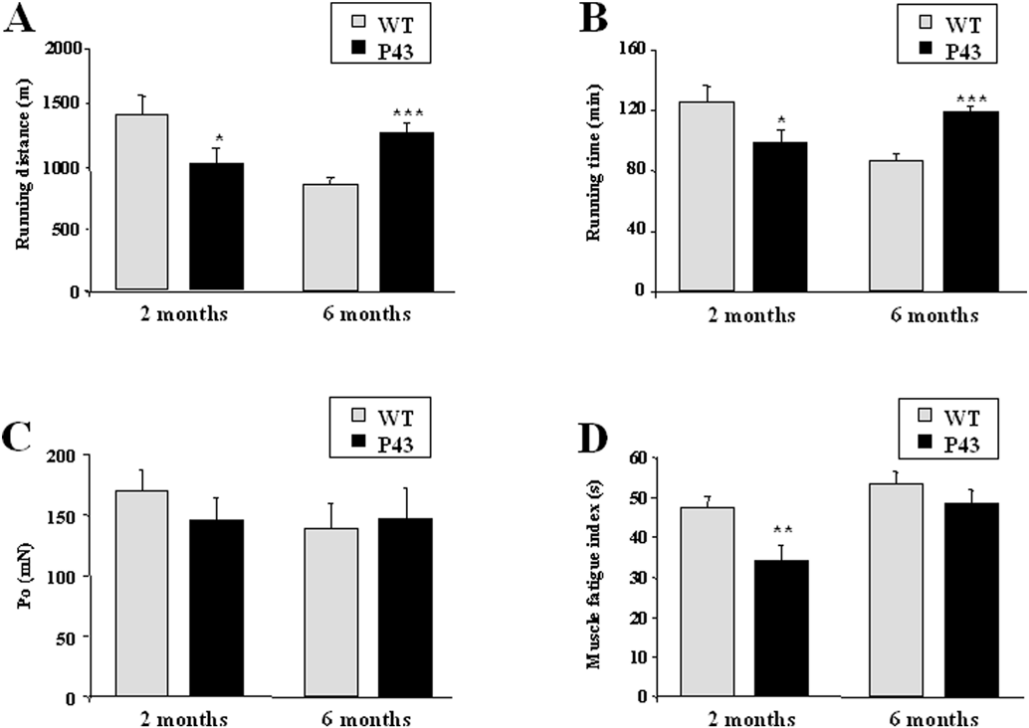
Influence of p43 overexpression on exercise endurance and on muscle contractile activities at 2 and 6 months of age. (A–B) 2 and 6 month-old transgenic mice (p43) and wild-type controls (WT) with similar body weights (n = 6 for each groups) were subjected to a forced treadmill exercise test. Running distance (A) and running time (B) are indicated. (C–D) Muscle contractile studies of 2 and 6 month-old transgenic mice (p43) and wild-type controls (WT) with similar body weights (n = 6 for each groups). Po (C) and Muscle fatigue index (D) are indicated. *p<0.05, **p<0.01 and ***p<0.001.

Muscle oxidative capacity is a crucial factor for determining endurance and fatigue. We thus compared exercise performance between untrained, body-weight-matched transgenic and wild-type mice as previously described [19]. Mice were run on treadmills until exhaustion. The running time and distance performed by control animal decreased from 2 to 6 months of life, in parallel to a rise in muscle fatigability (Figure 12). Just at the opposite, running time and distance performed by transgenic mice increased during this period, whereas muscle fatigability decreased (Figure 12). As a consequence, physical performances were significantly improved in 6 months old-p43 overexpressing mice, whereas they were altered in younger animals. Similarly, whereas fatigability was higher in 2 months-old transgenic mice, it was fully restored 4 months later.

From these results, it appears that the initial rise in mitochondrial activity recorded in p43 overexpressing mice induced long term consequences characterized by the occurrence of severe sarcopenia in relation to an enhanced expression of MAFbx and/or MuRF1 until 11 months. However, in older animals, this proteolytic pathway seems to be deactivated, thus suggesting that alteration of muscle protein synthesis is probably a major explanation for the important sarcopenia recorded in 23 months old mice.

## Discussion

### Influence of p43 overexpression on mitochondrial activity

We have previously shown that p43 overexpression in skeletal muscle of 2 month old mice increases mitochondrial transcription and mitochondrial biogenesis, hence leading to a stimulation of mitochondrial respiration [12]. To study the influence of this overexpression during aging, we have analyzed changes in mitochondrial activity and biogenesis in skeletal muscle at different ages: 2, 6, 11 and 23 months. We recorded a biphasic response of mitochondrial activity to p43 overexpression. First, mitochondrial biogenesis seems to be initially stimulated, as demonstrated by a rise in mitochondrial mass clearly apparent in histological studies (Fig. 2), in association with a transient increase in mtDNA levels. The initial increase in COX and CS activities clearly argues also in favour of a general stimulation of mitochondrial activity, also in agreement with the rise in mitochondrial respiration observed in the presence of succinate and rotenone. However, this transient stimulation is followed by an actual impairment of the organelle activity reflected by a decline in COX and CS activities, associated to a decrease in mitochondrial respiration under succinate rotenone and in mtDNA content observed from 2 or 6 months after birth.

Several other interesting features also appears in this study. Among them, we have shown *in vivo* that the maximal activity of complex II is poorly affected by p43 overexpression. In addition, we report here a selective increase in mitochondrial respiration measured in the presence of complex II substrates, occurring only in 2 months old transgenic mice. As Complex II maximal activity was not affected in these animals, such a result suggests that p43 improves the efficiency of this complex, considered as the second entry of the respiratory chain. One possible explanation could be that, despite the general increase in mitochondrial activity occurring in these younger animals, complex I activity remains unchanged, thus favouring the second entry of the respiratory chain. This possibility also well agrees with the observation that the rise in complex II-dependent respiration disappears in 6 months old animals, when maximal complex I activity is stimulated. In this hypothesis, this could reflect an adaptative change in response to the stimulation of the organelle activity. Other adaptative changes are also apparent with the observation that maximal activity of complex I displays opposite age-related changes to that recorded for COX or CS maximal activity.

### Influence of p43 overexpression on muscle oxidative stress

In order to understand the age-related decrease in mt-DNA content and several parameters of mitochondrial activity recorded in transgenic mice, we tested the possibility that this event could result from the occurrence of an oxidative stress, for the following reasons: i) mitochondria are the major intracellular source of Reactive oxygen species (ROS) production ; ii) *in vitr*o, p43 overexpression stimulates mitochondrial ROS production [13]; iii) mtDNA is a major target of ROS [20]. We found a significant rise in TBARS levels reflecting a higher rate of lipid peroxidation in 2 to 11 month old transgenic mice compared to wild types. In addition, we also observed a delayed decrease in protein thiols (SH groups) levels when compared to that recorded in control animals detected in 6 months old and older mice. This observation clearly reflected a stronger protein oxidation rate in muscle of p43 overexpressing mice. Interestingly, this oxidative stress takes place in spite of the induction of antioxidant defense systems. Antioxidant enzyme activity such as catalase and superoxide dismutase (SOD) which are up regulated against free radical damage, were transiently (SOD in 2 month old animals) or permanently (catalase) increased in quadriceps muscle. These results agree well with the study of UCP expression. One of the main adaptations of mitochondria to oxidative stress is a mild uncoupling of oxidative phosphorylation that reduces ROS mitochondrial production by lowering mitochondrial membrane potential [16], [17]. It has been shown that UCPs, by decreasing mitochondrial membrane potential, might attenuate cellular oxidative damage in some pathophysiological states leading to increased oxidative stress such as cancer cachexia [21]. Here we found that UCP2 and UCP3 mRNAs and proteins were strongly increased in quadriceps muscle of 2-month old transgenic mice relatively to control ones. These results were in line with a previous study showing that overexpression of p43 in human dermal fibroblasts stimulates ROS production and induced a strong decrease of mitochondrial membrane potential [13]. An activation of UCP2 and UCP3 gene expression associated with an increase of catalase activity has previously been reported in atrophied gastrocnemius muscles of tumour-bearing rats [22], [23]. However, this upregulation of UCPs expression was only recorded in 2 month old animals, suggesting that this protective mechanism is not permanently triggered during a long-term oxidative stress, a phenomenon partly explaining the progressive decline in mtDNA content occurring during aging of p43 overexpressing mice, despite the permanent upregulation of catalase activity.

### Influence of p43 overexpression on muscle development

As previously shown [12], p43 overexpression increased the expression of MyHC-IIa and MyHC-IIx in two different oxido-glycolytic muscles in younger mice, but decreased MyHC-IIb expression, thus inducing a shift toward the acquisition of a slow-twitch muscle phenotype. Interestingly, alterations of mitochondrial activity occurring during aging were associated with an important reduction in MyHC-I, IIx and IIb expression. Although remaining higher than in control animals, MyHC-IIa expression was significantly lower in 6 month old compared to 2 month old trangenic mice. These results suggest that p43-induced mitochondrial alterations led to a general reduction in MyHC expression, particularly MyHC-IIx. As myosins have to be considered as quantitatively major proteins in skeletal muscle, they also suggest that protein synthesis is altered in p43 overexpressing mice.

In agreement with the fact that oxidative stress is known to induce muscle atrophy, p43 overexpression induces a skeletal muscle loss detected since 6 months of age. After the initial shift in contractile phenotype observed in younger animals, as previously discussed, p43 overexpression clearly decreased all MyHC expression, particularly MyHC-IIx. In addition, as type IIX and IIB are the predominant fibers in quadriceps muscles, our data suggest that the reduced muscle weight in transgenic mice during aging results for its most part from the selective atrophy of fast-glycolytic fibers.

Muscle proteolysis is known to be mediated by the ubiquitin proteasome pathway via two muscle-specific ubiquitin ligases E3: Atrogin-1/MAFbx and MuRF1. In particular, MuRF1 overexpression results in a disruption of contractile proteins by interacting with titin [24]. Interestingly, the expression of these two critical regulators of muscle proteolysis was increased in quadriceps muscle of 2-months old transgenic mice compared to controls, at a time when muscle atrophy was not detected. However, muscles of 2 month old transgenic mice are richer in oxidative fibers known to be more resistant to atrophy [25]. At 6 months of age, when muscle atrophy became evident, only MAFbx expression was increased in p43 overexpressing mice. Interestingly, age-related changes in MAFbx and MuRF1 expression in transgenic mice displayed important differences, with predominant MAFbx overexpression in 6 month old animals, and a specific upregulation of MuRF1 expression in older animals. These results suggest that MAFbx and MuRF1 mRNA expression under atrophic conditions are induced via distinct signalling pathways. Over all, this set of data indicates that p43 overexpression induces skeletal muscle atrophy probably by stimulating the ubiquitin proteasome pathway via two muscle-specific ubiquitin ligases E3: MAFbx and MuRF1. However, the observation that the more important muscle loss occurs in 23 months old transgenic animals, at a time where expression levels of these ubiquitin ligases are restored also suggest that muscle proteosynthesis is severely impaired.

Experiments concerning muscle functionality have provided surprising data. Miura and co-workers have previously shown that 2-months old mice overexpressing PGC-1α ran less than wild-type mice because these transgenic mice presented a mitochondrial uncoupling and a marked decrease in the ATP muscle content [26]. Interestingly, in the treadmill test, the running time and distance performed by the 2-month old p43 overexpressing mice were decreased compared to wild-type. In addition, we observed a mitochondrial uncoupling attested by the strong increase of UCP2 and UCP3 expression at 2 month of age. These data suggest that p43 overexpression in young animals by increasing uncoupled oxidative phosphorylation caused ATP deprivation, resulting in a reduction of exercise perfomance as described in PGC-1α transgenic mice at the same age [26]. More surprising is the observation that despite the fact that muscle growth is reduced in 6 months old transgenic animals relatively to that recorded in control mice, p43 overexpression seems to improve muscle fatigability and endurance at this stage. This point is currently under investigation in order to study the metabolic influence of p43 overexpression, as changes in fuel metabolism could greatly influence animal endurance.

In conclusion, we demonstrated that p43 overexpression transiently stimulated mitochondrial biogenesis and activity. In turn, this influence leads to the occurrence of an oxidative stress probably at the origin of muscle sarcopenia. Interestingly, a similar muscle wasting has been described in mice overexpressing PGC-1α [26]. Hence, they underline the importance of a tight control of p43 expression and suggest that its deregulation could be involved in some mitochondrial diseases and could be one of the parameters involved in the occurrence of sarcopenia.

## Methods

### Animals

All animals experiments were performed according to European directives (86/609/CEE) and approved by Comité d'Ethique en matière d'Expérimentation Animale: Région Languedoc-Roussillon. The transgenic mice were generated as previously described [12].

### Histological analysis

Fresh muscles were immersed in a solution of 3.5% glutaraldehyde in phosphate buffer (0.1 M, pH 7.4) overnight at 4°C. They were then rinced in phosphate buffer and post-fixed (1% osmic acid, 0.8% potassium ferrocianide) for 2 h in the dark and at room temperature. After two rinces in a phosphate buffer, muscles were dehydrated in a graded series of ethanol solutions (30–100%). The cells were embedded in EmBed 812 DER 736. Thin sections (85 nm ; Leica-Reichert Ultracut E) were collected at different levels of each block. These sections were counterstained with uranyl acetate and lead citrate and observed using a Hitachi 7100 transmission electron microscope in the Centre de Ressources en Imagerie Cellulaire de Montpellier (France).

### Protein studies

50 µg of mitochondrial extracts or total protein extacts were electrophoresed onto 10% SDS-Page gels and blotted onto PDVF membranes. The presence of p43 was assessed using RHTII antisera as previously described [7]. The presence of α-tubulin used for normalization was assessed using a monoclonal anti-α-tubulin (T5168, SIGMA). The presence of UCP2-3 was assessed using a monoclonal anti-UCP2 which also detects UCP3 (sc-6525, Santa Cruz). The presence of Cu/Zn -SOD and Mn- SOD was assessed using antibodies respectively from Biovalley and Labfrontier. The presence of proteins were revealed using a chemioluminescent Western blot procedure (ECF kit, Amersham) and analyzed with a PhosphorImager (Molecular dynamics).

### Gene expression studies

Total RNA were isolated from quadriceps muscle using the Trizol method (invitrogen). Samples were reverse transcribed using superScript first-Strand synthesis System (invitrogen), and quantitative PCR reactions were performed on the cDNAs in the presence of fluorescent dye (SYBR Green, Bio-Rad). The following primers were used: UCP2 (forward, GCATTGGCCTCTACGACTCTG ; reverse, AGCGGACCTTTACCACATCTG); UCP3 (forward, GACCCACGGCCTTCTACAAA ; reverse, ATTCCCGCAGTACCTGGACTT) ; MAFbx (forward, GACTGGACTTCTCGACTGCC ; reverse, TCAGCCTCTGCATGATGTTC) ; MuRF1 (forward, CAACCTGTGCCGCAAGTG; reverse, CAACCTCGT GCCTACAAGATG) ; MyHC-I (forward, CCTTGGCACCAATGTCCCGGCTC ; reverse, GAAGCGCAATGCAGAGTCGGTG); MyHC-IIa (forward, ATGAGCTCCGACGCCGAG; reverse, TCTGTTAGCATGAACTGGTAGGCG); MyHC-IIx (forward, AAGGAGCAGGACACCAGCGCCCA; reverse, ATCTCTTTGGTCACTTTCCTGCT); MyHC-IIb (forward, GTGATTTCTCCTGTCACCTCTC; reverse, GGAGGACCGCAAGAACGTGCTGA) and RPS9 (forward, CGGCCCGGGAGCTGTTGACG ; reverse, CTGCTTGCGGACCCTAATGT). DNA product of the expected size was confirmed for each primer pair. After normalization by RPS9, all results are expressed as percent of control as means±SEM. Student's t-test was used to determine all p values.

### Measurement of mtDNA copy number

The mtDNA content is the mtDNA copy number normalized to the copy number of a nuclear gene. After extraction of muscle DNA, quantitative PCR reactions were performed using ND5 (forward, GGCAGACGAACAAGACATCCGAAA; reverse, GCTAGGCGTTTGATTGGGTT) for mtDNA copy estimation, and 18S for the nuclear genome. All results are expressed as percent of control as means±SEM. Student's t-test was used to determine all p values.

### Enzymatic activities of mitochondrial complexes

Muscle enzyme activites were measured from whole gastrocnemius homogenates. Proteins concentration was measured using the Bio-Rad protein assays kit. Citrate synthase activity was measured as described [27]. Cytochrome oxidase was measured as described [28] and expressed in mU/mg protein.

### Mitochondrial respiration on isolated fibers

Saponin-permeabilized muscle fibers were prepared from Gastrocnemius as previously described [29]. Briefly, thin fibre bundles were excised along the fiber orientation to avoid mechanical damage to the cells. Fibers were carefully separated from each other using sharp-ended forceps and needles in cooled solution A (containing in mM: CaK_2_EGTA 2.77, K_2_EGTA 7.23, MgCl_2_ 6.56, DTT 0.5, potassium 2-(*N*-morpholino) ethansulphonate (K-Mes) 50, imidazole 20, taurine 20, ATP 5.3, phosphocreatine 15, pH 7.1 adjusted at 4°C, free Ca^2+^ concentration 0.1 µM, a condition which prevents contraction of the bundles) until bundles of roughly 20–30 fibers were obtained with only small areas of contact between them. Fibers were then permeabilized by incubation in solution A supplemented with 50 µg ml^−1^ saponin with gentle shaking for 30 min at 4°C. To completely remove all metabolites, including trace amounts of ADP, fibres were washed three times in respiration solution B (containing in mM: CaK_2_EGTA 2.77, K_2_EGTA 7.23, MgCl_2_ 1.38, DTT 0.5, K-Mes 100, imidazole 20, taurine 20, and K_2_HPO_4_ 3, pH 7.1 adjusted at 25°C, free Ca^2+^ concentration 0.1 µM) supplemented with bovine serum albumin (2 mg ml^−1^). Respiratory parameters of fiber bundles (20–30 fibers) were recorded at 25°C using a Oroboros oxygraph. Resting respiration (*V*_0_) was initiated in the presence of complex I (5 mM malate and 5 mM pyruvate) or complex II substrates (10 mM succinate and 2.5 µM rotenone), and maximal ADP-stimulated respiration was measured with one addition of saturating ADP concentration (1 mM)(*V*_max_).

### Determination of Muscle lipid peroxidation and thiol (SH-group) levels

The concentration of lipid peroxide was estimated by the thiobarbituric acid-reactive substances (TBARS) method using total extracts from quadriceps muscle as previously described [30]. The concentration of Thiol levels using total extracts from quadriceps muscle was measured as previously described [31].

### Treadmill studies

Treadmill studies were performed as previously described [19]. Prior to the exercise performance test, the mice were accustomed to the treadmill (Bioseb) with a 5 minutes run at 7 m/min once per day for 2 days. The exercise test regimen was 10 m/min for the first 60 minutes, followed by 1 m/min increment increases at 15 minutes intervals. Exhaustion was defined when mice were unable to avoid repetitive electrical shocks.

### Muscle contractile studies

Animals were anesthetized with pentobarbital sodium. The EDL (fast twitch) muscle from the right hindlimb were surgically exposed. The excised muscles were immediately placed into a custom-built plexigas chamber filled with Krebs-Ringer solution [composition (in mM): NaCl 11.9, Kcl 0.5, CaCl_2_ 0.125, MgSO_4_ 0.1 KH_2_PO_4_ 0.1, Glucose 1, NaHCO_3_ 2.5, Mannitol 0.11] equilibrated with 95% O_2_- 5% CO_2_ gas and maintained at 25°C, pH 7.4, which is optimal for maintaining the viability of the muscles in vitro for the duration of the experimental protocol [32], [33]. Contractile properties of the EDL was assessed *in vitro* according to the methods described in detail previously [33], [34]. After a 15-min equilibration in the bath, the muscle was connected to an isotonic force transducer (model 305B, Cambridge instruments, Aurora Scientific Inc, Ontario, Canada) and was stimulated along its entire length with platinum wire electrodes. Criswell et al., have previously demonstrated that this method of stimulation results in optimal muscle stimulation, compared with direct stimulation using stainless steel electrodes [35]. Optimal length, i.e the length producing maximal twitch tension, was determined. All subsequent measurements were made at optimal length. The tension-frequency response was then determined (701B Stimulator, Aurora Scientific Inc, Ontario, Canada) using stimulations trains of 500-ms at frequencies of 1–100 hz. Stimulus trains were separated by a 1 min interval. Maximal isometric tetanic tension (Po) were then determined. Three minutes after the tension-frequency determination, fatigue resistance was evaluated using a low-frequency fatigue protocol of 50 hz trains of 700-ms delivered every 2 s for 5 min [36]. After all measurements, the muscle was removed from the bath, trimmed of connective tissue, blotted dry and weighed on a analytical balance. Po was co-expressed in mN or mN/mg. Muscle fatigue index (Tlim) was defined as the time taken for a 50% reduction in maximal power output. Values are expressed as mean±SEM. Variables were compared between groups using analysis of variance ANOVA, and Newmann Keuls *post-hoc* multiple comparison procedure where significance was detected. Signifiance was set at *p*<0.05.

### Statistical analyses

All results are presented as means±SEM, or as percentages. The significance of the difference between groups was evaluated with Student's t-test. *p<0.05; **p<0.01; ***p<0.001. p<0.05 was considered significant.
